# Biological research involving people with lived experience of childhood trauma: a trauma-informed approach

**DOI:** 10.3389/fpsyg.2026.1788726

**Published:** 2026-03-06

**Authors:** Mica Komarnyckyj, Derek Clougher, Bethany Thompson

**Affiliations:** 1 Biomedical Research Centre, Division of Psychology & Mental Health, University of Manchester, Manchester, United Kingdom; 2 Bipolar and Depressive Disorders Unit, Hospital Clínic de Barcelona, ISCIII, Barcelona, Spain; 3 Manchester University NHS Foundation Trust, Manchester, United Kingdom

**Keywords:** ACEs (adverse childhood experiences), biological psychiatry, childhood maltreatment, childhood trauma, neuroimaging, neuroscience, trauma, trauma-informed

## Abstract

In this Perspective, we highlight the growing need for trauma-informed practices as researchers increasingly work across disciplines to study the effects of childhood trauma. We emphasise that trauma-informed principles must be embedded not only to protect participants, but also to safeguard interdisciplinary researchers conducting biological psychology and psychiatry research with people who have lived experience of trauma. We offer practical recommendations and ethical considerations for minimising risk during trauma research, together with example content for trauma-informed researcher training. We uncovered an unmet need to systematically review what training is already available and identify the training and knowledge gaps that exist within trauma research. Understanding these realities will help shape practical, evidence-based approaches that support both participants and researchers.

## Introduction

This perspective covers the ethical and practical challenges of conducting biological research with people who have lived experienced of childhood trauma. Over the past two decades there has been a sustained rise in childhood trauma research - encompassing maltreatment, abuse, neglect and adverse childhood experiences (ACEs) - across many disciplines such as psychology, psychiatry, biology and neuroscience. (Sindhura and Ganesh Kumar, 2025; Tran et al., 2018). This growth in scientific focus emphasises the need to raise awareness of how trauma-informed principles can be embedded in research, not only to protect participants, but also to safeguard interdisciplinary researchers (SAMHSA, 2014; Dhollande et al., 2025).

In the context of research, we translate the *“trauma-informed”* framework—which originated within clinical and social care settings—to mean: (1) recognising the lasting impact of traumatic experiences and how these may affect individuals during research participation; (2) considering the potential psychological impact for researchers working with individuals who have experienced trauma; and (3) combining institution-wide mechanisms, with research design and implementation strategies to prevent re-traumatisation for participants and adverse psychological impacts for researchers (Dhollande et al., 2025; OHID, 2022; SAMHSA, 2014).

Converging evidence indicates that childhood trauma can impact the brain, immune system and daily functioning later in life (McLaughlin et al., 2020; Ross et al., 2021; Danese and Lewis, 2017; Torres-Berrío et al., 2025). Experiences of trauma during this critical developmental window confers transdiagnostic risk for a broad range of mental health difficulties and increases the risk of psychiatric comorbidity (van Nierop et al., 2015). There is increasing emphasis on transdiagnostic models of mental health aiming to advance psychiatric treatment by identifying common biological mechanisms across disorders, which could be targeted in comorbidity and trauma-focused interventions (McLaughlin et al., 2020).

Given the complex aetiology of disorders, identifying transdiagnostic mechanisms of childhood trauma is an interdisciplinary challenge. Addressing such research questions relies on collaboration from biological researchers trained outside of clinical psychology or psychiatry disciplines, such as neuroscience (Ross et al., 2021), immunology (Danese and Lewis, 2017) and neurobiology (Torres-Berrío et al., 2025). Concerningly, a recent study highlighted the urgent need for trauma-informed training, with only 5% of American Psychological Association accredited clinical psychology doctoral programmes requiring a course related to trauma-informed care (Foltz et al., 2023). These numbers are likely even lower in biological courses, where the focus is on technical and research skills rather than supporting clinical practice. Notably, our desk-based review uncovered that this training need has not yet been systematically investigated.

Consequently, many researchers without trauma-focused training are likely to now work in research roles directly with people who have experienced trauma and/or analyse trauma-related data. Project planning often focuses on the protection of participants, while the emotional toll on researchers receives less attention (Berger, 2021; Dhollande et al., 2025; Kiyimba and O’Reilly, 2016). Furthermore, formal trauma-informed training and structures preventing indirect trauma (e.g., vicarious trauma and secondary traumatic stress) are not typically available at institutions (Dhollande et al., 2025; Berger, 2021).

In this perspective, we provide recommendations for minimising the risks associated with interdisciplinary research that is investigating the biological mechanisms of childhood trauma. Our recommendations support participant and researcher well-being and are broadly applicable across mental health and trauma research. We draw upon best practice from clinical psychology and psychiatry, and literature on vicarious trauma in nursing (Dhollande et al., 2025; Taylor et al., 2016) and qualitative research (Kiyimba and O’Reilly, 2016; Alessi and Kahn, 2023).

## Participant protection: trauma-informed research environments

Institutions conducting trauma-related research have an ethical and scientific responsibility to safeguard participants against re-traumatisation and researchers against indirect trauma. A trauma-informed research environment recognises and proactively manages the potential impact of trauma research. Furthermore, it has policies and procedures which prevent psychological harm, whilst promoting safety, trust, respect, and emotional support for everyone involved.

**Figure 1:**
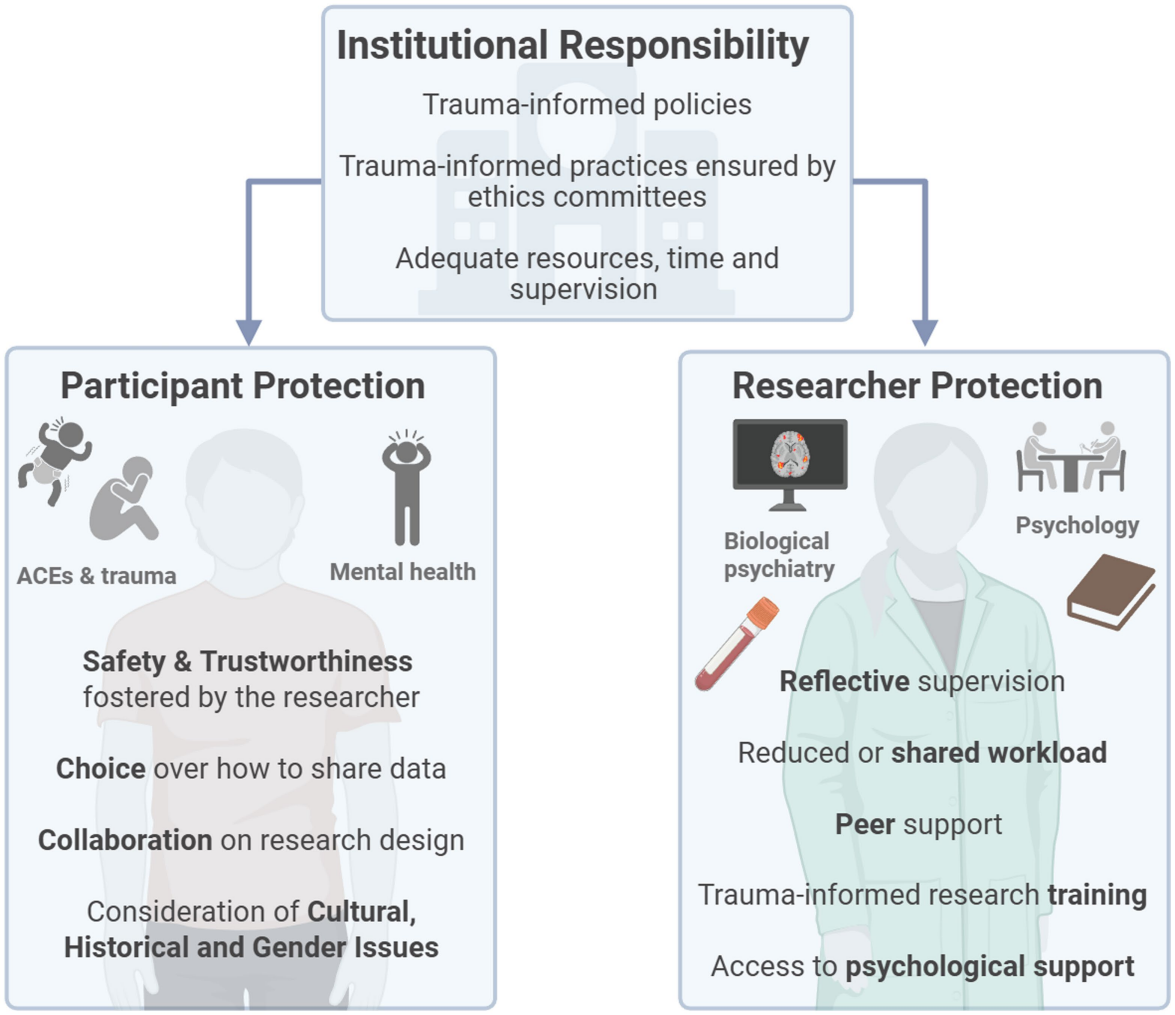
Researching trauma with care: recommendations for protecting researcher and participant well-being. ACEs, adverse childhood experiences. Created in BioRender (Komarnyckyj, 2026, https://www.biorender.com/exawbdw).

Researcher training in the six trauma-informed principles is critical: Safety; Trustworthiness; Peer Support; Collaboration and Mutuality; Empowerment, Voice, and Choice; and Cultural, Historical and Gender Issues (SAMHSA, 2014) (Figure 1). These important concepts, originally developed for clinical practice, must be translated into research environments, to ensure researchers consider the impact of their work on both participants and themselves, and minimise the risk of re-traumatisation (Isobel, 2021).

For researchers, particularly non-clinicians, it can be challenging to know how to respond appropriately when participants recall traumatic experiences. To reduce institutional costs associated with trauma-informed training, it could be delivered as pre-recorded online training modules with integrated assessments to confirm understanding. Trauma-informed training for research settings is not yet widely available. We propose that it should be co-created in collaboration with researchers, training specialists, clinicians and people with lived experience of trauma and then be disseminated across the research community.

Online training would reinforce the six trauma-informed principles throughout. Example modules may include one on *Trauma-Informed Communication*, recognising and responding to distress as non-clinicians (Alessi and Kahn, 2023). Teaching on communication skills such as active listening and the therapeutic principle of “unconditional positive regard” would be included (Rogers, 1959). Tips on recognising early signs of participant distress and how to respond to help participants feel supported and minimise risk of re-traumatisation would also be covered. One such example is demonstrating to researchers how they can teach grounding techniques to participants. This would empower them to support participants through distress and increase feelings of *“Safety”* during research visits (Alessi and Kahn, 2023).

Building on this, a module on *Risk and Safeguarding* procedures would also support researchers to protect the well-being of participants. This would include a local procedure for researchers to follow when they are concerned that a participant is an immediate risk to themselves or others. Additionally, it should emphasise identifying the aspects of the protocol that are more likely to be intrusive or distressing and ensuring the protocol remains flexible. To uphold the principle of “*Empowerment, Voice and Choice*”, this would include regularly reiterating to participants that they can pause, reschedule or decline any procedure at any point (Alessi and Kahn, 2023). This is particularly important given that routine medical procedures, such as blood tests, which involve close physical contact, or brain imaging, which involves a claustrophobic scanning environment, may feel more intrusive or distressing for individuals with a lived experience of trauma.

Participants should also be given the choice of how, when and what format (e.g., online vs. face-to-face) they report sensitive information regarding their previous trauma experience. Such options accommodate the individual needs of participants, thus respecting their “*Voice and Choice*” and fostering a sense of control and *“Safety.”* Both this module and the first could include animated and/or pre-recorded video examples of good versus bad practice to aid understanding.

The “*Collaboration and Mutuality”* and *“Peer Support”* principles could be incorporated into a module focused on *Lived Experience* & *Participant Involvement/Engagement*. This module would emphasise the importance of engaging people with lived experience when generating research ideas, designing study protocols, gaining feedback on project delivery and interpreting results—formally termed Participant and Patient Involvement & Engagement (Isobel, 2021). Through use of qualitative research extracts and composite narratives, this module would help researchers understand the potential long-lasting impacts of trauma on individuals and the different forms of trauma which may occur over a person’s lifetime (Willis, 2019). Training on consideration of “*Cultural, Historical and Gender Issues*” would also be embedded to ensure diverse perspectives are incorporated into project designs and that participant samples include underserved groups. For example, individuals who are from marginalised communities who experience historical oppression are often excluded from research due to systemic barriers. Disproportionately high rates of trauma are experienced within these groups, making their inclusion essential for equity and scientific validity (Charles-Rodriguez et al., 2025).

## Researcher protection: safeguarding against indirect trauma and burnout

It is essential that training incorporates a module on the *Protection of Researchers’ Well-Being*, including preventing and recognising signs of burnout within oneself and advice on promptly seeking appropriate help from healthcare services and the employing institution. It would cover self-protective and self-help strategies, such as engaging in regular hobbies, exercise and mindfulness. This module would, however, also highlight that whilst these strategies can be helpful, protection of researchers from occupational stress is not solely their own responsibility. Training should aim to support researchers with self-advocacy such as the skills to communicate their needs and set boundaries. Giving them the confidence and agency to raise work-related concerns with their supervisors and/or healthcare professionals before stress or burnout occurs. The responsibilities of the institution to provide safeguards and support are discussed in more detail in the following sections.

Researchers conducting interviews regarding mental health or past childhood trauma may hear disturbing first-hand accounts of trauma or its impact and witness participants’ emotional distress (Kiyimba and O’Reilly, 2016). Examples which may be delivered by non-clinicians during research include the Structured Clinical Interview for DSM-5 Disorders (SCID-5), Flexible Interview for ICD-11 (FLII-11) and any trauma or life events interview. Researchers delivering such interviews are at high risk of indirect trauma, which can manifest as fatigue, emotional numbness, intrusive imagery, social withdrawal and sleep problems (Dhollande et al., 2025; Berger, 2021). Such symptoms can have far-reaching consequences across personal and professional lives. Commonly, data collection is the responsibility of early career researchers (e.g., PhD students, post-doctoral researchers and research assistants) and therefore the risk may be exacerbated due to inexperience and potential lack of trauma-informed training.

Aligned with recommendations for clinical psychology practice, an essential safeguard for those conducting in-depth interviews with trauma-affected individuals is reflective supervision, regardless of academic seniority or career stage. This should be delivered by a clinician or academic supervisor who is experienced in working with populations who have experienced trauma and has in-depth knowledge of trauma-informed research. The frequency of supervision should be determined on a case-by-case basis, considering the frequency and intensity of the interviews and the experience of the researcher.

Reflective supervision provides dedicated time, outside of operational or scientific meetings, for researchers to share and process feelings confidentially, raise concerns and seek advice about future interactions with participants. An effective supervisor can identify early signs of distress and emotional burden and will continuously assess the potential risk for burnout. In doing so, they can proactively offer solutions before harm is caused. For example, this may include offering adaptations to the work schedule, promoting self-care strategies, or making referrals for psychological support.

Where required, researchers conducting in-depth interviews should have access to specialised therapy that is tailored to the unique impact of their work, rather than relying on generalised low-intensity therapy commonly available for staff and students. Incorporating group supervisory sessions can also be beneficial, as this gives colleagues the opportunity to support and connect with one another, share experiences and coping strategies and normalise emotional responses through empathy. This can promote well-being and reduce feelings of emotional isolation which may occur as trauma-related work is often conducted independently (Dhollande et al., 2025).

Outside of interviews, other research activities may present risk for indirect trauma, including administering trauma questionnaires (e.g., Childhood Trauma questionnaire, Adverse Childhood Experience Questionnaire, Life Events Checklist for DSM-5) and analysing transcripts which contain trauma narratives. Unlike healthcare professionals, researchers may not have the experience or resources to intervene or offer therapeutic support, which can lead to feelings of helplessness, frustration and despair (Dhollande et al., 2025; Taylor et al., 2016; Kiyimba and O’Reilly, 2016; Alessi and Kahn, 2023). Even those researchers who never interact directly with participants but immerse themselves deeply in understanding the long-term effects of trauma can feel the emotional weight of this work. Globally, around one-fifth of people experience at least one adverse childhood event, meaning this research can also be personally triggering (Madigan et al., 2025). Institutions should encourage supervisors to regularly check in with all team members about how trauma-related research is affecting them.

A familiar challenge for researchers is limited budgets and time constraints, often leading to unmanageable workloads. When the topic under investigation is emotionally demanding, like trauma or mental health, this exacerbates the risk of work-related stress and burnout. Frequent repeated exposure to distressing information may also cause compassion fatigue, with symptoms including irritability and decreased empathy, having negative impacts both for the researcher and participant (Bennett et al., 2025). Research planning must account for the psychological impact of this work, with expectations on participant recruitment reduced compared with lower-risk projects. Emotionally demanding activities should be shared across team members with different levels of experience, rather than being the responsibility of a single researcher. This minimises the burden of the work and facilitates informal peer support and shared reflection.

## Ethical oversight for trauma-informed research

It is imperative that trauma-informed research practices are considered during the conceptualisation and design of research projects. Only by reflecting on potential impacts early, can research teams ensure adequate resources, training and suitably experienced team members are in place. Furthermore, input from lived experience collaborators during these initial phases of a project will help ensure the communities’ priorities and needs are put at the forefront of research design.

Ethics Committees and Institutional Review Boards (IRBs) have a critical role in ensuring core harm prevention strategies are integrated before granting ethical approval. They must assess whether research applications include: an evaluation of risk for participants (e.g., distress or re-traumatisation) and researchers (e.g., burnout or indirect trauma); plans for monitoring and reducing the impact of the research (e.g., training in trauma-informed practices, adequate supervision, reasonable workloads and timescales); and processes for addressing problems should they arise (e.g., participant distress protocols and resources for psychological support) (Dhollande et al., 2025).

Once a project has started, senior research staff and supervisors are responsible for ongoing ethical oversight. Critically, they must anticipate potential problems before they arise and adapt protocols as needed to mitigate against emerging risks. Ultimately trauma-informed research requires flexible and responsive researchers working with Ethics Committees to support safety and well-being.

## Broad applicability for trauma & mental health research

This perspective has focused on biological research relating to childhood trauma due to increased scientific focus on this area, and strong likelihood that research will cover sensitive, personal and potentially triggering activities (Sindhura and Ganesh Kumar, 2025; Tran et al., 2018). However, parental experiences prior to birth may also give rise to trauma, presenting transdiagnostic risk for later life mental health and developmental difficulties. Prenatal trauma can occur when a mother experiences abuse/violence victimisation, stress, anxiety or substance dependence during pregnancy. These exposures in the womb have been associated with physical, social, cognitive and emotional problems in offspring (Tung et al., 2024; Talge et al., 2007; Streissguth et al., 1994). There is also increased risk of childhood trauma in offspring due to unstable or unsafe family environments (Cohodes et al., 2019).

Trauma can also have intergenerational effects. Exposure to severe or chronic stress or trauma (e.g., war, poverty and ACEs) may become biologically embedded through epigenetic alterations. These are long-term changes to gene expression via mechanisms that do not alter the DNA sequence directly but can be inherited by offspring (Tuulari et al., 2025). These changes are associated with microscopic neuropathological and inflammatory changes that may alter neural functioning, hypothalamic–pituitary axis activity and immune functioning (Yoshino and Dwivedi, 2020; Nelson et al., 2020; Berens et al., 2017), increasing the risk of mental illness in individuals who have experienced trauma and their descendants (Mert et al., 2025; Pilkay et al., 2024).

Researchers are therefore encouraged to employ trauma-informed approaches which take into account the lasting impact of parental experiences and potential for co-occurrence between multiple forms of adversity. For example, Indigenous populations who experience higher rates of ACEs, historical trauma, racism and systemic discrimination, may be at increased risk for trauma across multiple contexts and generations (Radford et al., 2022).

Notably, a core tenet of “trauma-informed practice” when applied within clinical and social care settings, is working with an assumption that any person accessing support—for example emergency healthcare, substance use and mental health services—may have been impacted by trauma, irrespective of trauma disclosure (Greenwald et al., 2023). Building on this, we argue that our recommendations are broadly applicable across all mental health research, even when the focus is not trauma directly, given the high prevalence of trauma and severe personal distress (e.g., self-harm, suicide ideation) in people who experience psychiatric conditions.

## Future directions

We would like to highlight that trauma and mental health research can be deeply meaningful for both participants and researchers and does not inevitably cause harm when appropriate safeguards are in place. When conducted with sensitivity and adequate support, it can empower participants and give researchers a strong sense of purpose and fulfilment.

Despite growing attention to trauma-informed principles in government policy and healthcare services, there remains limited understanding of how these principles are being implemented in biological psychology and psychiatry research settings. Our review of the literature highlighted that very little is currently known about the level of trauma-informed training being provided to interdisciplinary researchers investigating the long-term effects of childhood or adult trauma on the brain, behavior and physical or mental health. There is a critical need within this field to systematically review what training exists, identify training and knowledge gaps and listen to researchers’ lived experience of working with people who have experienced trauma. Understanding these realities will help shape practical, evidence-based approaches that support both participants and researchers.

We recognise the need for researcher self-care strategies to manage the day-to-day impact of trauma-related research (Berger, 2021). But without the institutional and supervisory level support we have recommended, many researchers may not have the capacity, energy or time to integrate these practices effectively. By framing well-being as a shared responsibility between individuals and institutions, we can foster a safer and more sustainable research culture that supports high-quality and ethical science.

In this perspective, we provide initial recommendations for safeguarding well-being within interdisciplinary teams working on trauma and mental health related projects. Moving forward, formalised guidelines and standardised training are essential for people working in this field, which must be built from genuine collaboration between people with lived experience, clinicians, institutions and researchers.

## Data Availability

The original contributions presented in the study are included in the article, further inquiries can be directed to the corresponding author.
